# Spatial Disparity and Influencing Factors of Coupling Coordination Development of Economy–Environment–Tourism–Traffic: A Case Study in the Middle Reaches of Yangtze River Urban Agglomerations

**DOI:** 10.3390/ijerph18157947

**Published:** 2021-07-27

**Authors:** Qian Chen, Yuzhe Bi, Jiangfeng Li

**Affiliations:** Department of Land Resource Management, School of Public Administration, China University of Geosciences, Wuhan 430074, China; chenqian@cug.edu.cn (Q.C.); biyuzhe1998@163.com (Y.B.)

**Keywords:** economy–environment–tourism–traffic system, coupling coordination degree, exploratory spatial data analysis model, grey correlation degree analysis, the middle reaches of Yangtze river urban agglomerations

## Abstract

In the process of rapid development of economic globalization and regional integration, the importance of urban agglomeration has become increasingly prominent. It is not only the main carrier for countries and regions to participate in international competition, but also the main place to promote regional coordination and sustainable development. Coordinated economic, environmental, tourism and traffic development is very necessary for sustainable regional development. However, the existing literature lacks research on coupling coordination of the Economy–Environment–Tourism–Traffic (EETT) system in urban agglomeration. In this study, in order to fill this gap, we establish the index system from four dimensions of economy, environment, tourism and traffic, and select the influencing factors from the natural and human perspectives to exam the spatio-temporal changes and influencing factors in the coupling coordination of the EETT system using an integrated method in the Middle Reaches of Yangtze River Urban Agglomerations (MRYRUA), China. The results indicate that the coupling coordination degree of the EETT system transitioned from the uncoordinated period to the coordinated period, while it showed an increasing trend on the whole from 1995 to 2017. The spatial agglomeration effect has been positive since 2010, while “High–High” and “Low–High” agglomeration regions were transferred from the east to the south. Land used for urban construction as a percentage of the urban area and vegetation index has a great impact on the coupling coordination degree. These results provide important guidance for the formulation of integration and coordinated development policy in the MRYRUA, and then increase China’s international competitiveness by improving the contribution of urban agglomerations to GDP.

## 1. Introduction

The coordination degree of a coupling system plays an important role in regional stability and sustainable development. With the gradual introduction of the integrated development policy of traffic and tourism, eco-tourism development planning, eco-economic policies and other strategic laws and regulations, the trend and importance of the integrated development of economy, environment, tourism and traffic are highlighted from the national to the social level [1,2,3]. Although the development of regional economy and tourism will bring huge economic and social benefits, at the same time, it may also cause damage to the natural environment and increase the burden of transportation [4,5,6]. When the environmental deterioration breaks through its threshold, it will increase the cost of economic activities and restrict the long-term development of regional tourism [7,8]. Meanwhile, unbalanced development of tourism, economy, environment and traffic has resulted in various problems, such as the resource curse phenomenon, imbalance between tourism and traffic growth, potential environmental hazards caused by economic, traffic and tourism development, etc. [9,10]. Academics have shown considerable interest in conducting research on the relationship or interaction among any two or three subsystems of tourism, economy, environment and traffic, and the research timescale mostly focuses on cross-sectional data or a short-term period [11,12]. However, there is a lack of overarching research on the coupling coordination development of the four subsystems, space analysis and the identification of its influencing factors. Therefore, it is of great significance for the realization of sustainable regional development to quantitatively analyze the Economy–Environment–Tourism–Traffic (EETT) coupling coordination relationship.

The Middle Reaches of Yangtze River Urban Agglomerations (MRYRUA) is the first approved national urban agglomeration in China and the second largest urban agglomeration in China. Under the background of the national regional coordinated development strategy and the strategy of the rise of central China, as an important part of the Yangtze River economic belt, the MRYRUA undertakes the role of connecting the east with the west and connecting the south with the north in terms of economic and transportation development, and is the core hub of the central and western regions connecting the Yangtze River Delta and Guangdong, Hong Kong and Macao [13,14]. At the same time, the MRYRUA guards the 955 km Yangtze River waterway, with the confluence of many tributaries. Its environmental conditions directly affect the environmental quality of the Yangtze River Basin, especially the lower reaches of the Yangtze River [15,16]. Since 1995, the MRYRUA has developed rapidly, forming a large metropolitan area. At the same time, the economy, transportation, and tourism have developed vigorously, which has caused certain pressure on the environment. If not guided scientifically, it may cause environmental damage to the whole Yangtze River Basin in the future due to exceeding the ecological carrying capacity [17,18]. The research on the spatiotemporal evolution and its influencing factors of coupling coordination degree in MRYRUA will not only help to formulate policies and guidelines for sustainable development, providing a reference for the land and space planning of urban agglomeration, but also provide ideas for the coordinated development of other urban agglomerations in China.

Economy, environment, tourism and traffic are important elements of regional development, and their development status is related to the sustainable development of the region [4,19,20]. Therefore, it is necessary to discuss the coordinated development status and changes of EETT as a whole, and to explore the influencing factors of coupling coordination status, which can provide ideas for solving the development problems caused by the imbalance between various systems. The coupling coordination degree model is an important model to clarify the coordination relationship among systems, which can be used to analyze the coupling and coordination relationship among the four subsystems of EETT; Exploratory spatial data analysis model is an important spatial analysis model, which is used to explore the spatial correlation of coupling coordination degree of different regions; The grey correlation degree analysis is the main model to analyze the importance of influencing factors, which can be used to explore the correlation between factors. The research on the evolution and impact mechanism of coupling coordinated development of EETT can provide scientific references for the rational allocation of resources, industrial transformation and integrated development of urban agglomerations. To this end, this study set three research objectives as follows: (1) to show the spatial pattern in the development of the coupling of the EETT system; (2) to identify the indicators influencing the coordination development of the four subsystems; and (3) to provide references for the more coordinated development of the EETT system during the innovation of regional policy.

## 2. Literature Review

### 2.1. Coupling and Coordinated Development of Economy, Environment, Tourism and Traffic

The research on the interactive relationship between tourism, ecology and economy originated earlier, and related research is relatively rich. For example, Chi-OK Oh [21] made an empirical analysis on the balance between tourism and economic growth in South Korea. Thapa et al. [22] believed that tourism development has both positive and negative forces on the ecological environment. Day J et al. [23] analyzed the challenges of energy and environment in the United States and China to the sustainable development of regional tourism. Lacitignola and Petrosillo [24] established a model of social–ecological coordinated development and vulnerability based on tourism. Patterson et al. [25] used the ecological footprint method to discuss the coordinated development of tourism environment.

In recent years, compared to research from a binary perspective, the systematic study of any three components is becoming a critical concern. A number of studies have focused on analyzing and forecasting the coupling coordination development of the economy, environment, and tourism, whether at province scale or city scale. With the rapid development of transport networks, some scholars have carried out studies on the coordination development of tourism, traffic and environment based on panel data [20,26]. A growing number of studies have indicated that regional economic mobility and transport investment are related to each other [12,27]. Transportation energy consumption growth and economic growth will reduce the environmental quality [5,20,28]. Tourism capacity, tourism carrying capacity and ecological change have an impact on the tourism industry [29,30]. Concerning the research methods, information entropy weight or analytic hierarchy processes have been used to determine the weight [28,31]. Grey correlation analysis has been used to establish the measurement model [32,33]. The coupling coordination degree model, autoregressive conditional heteroskedasticity and the environmental Kuznets curve model have been used to analyze the relationship between systems [4,28,34]. Global and local–spatial autocorrelation, identification of clusters, space Markov chain and Getis-Ord Gi* have been used to analyze the spatial relationship [19,35,36]. These related studies have revealed the relationships and influence mechanism in coupled systems, which provides the basis of the theoretical framework and methods for the study of the coupling relationship of EETT.

### 2.2. Influencing Factors on Coupling and Coordinated Development

There are many substantial and useful previous explorations in the research of influencing factors on coupling coordination degree. In recent years, attention has been paid to the analysis of a specific factor, and mostly adopted the method of econometric analysis. Pham [37] discussed the short-term and long-term effects of economic, social and energy factors on environmental degradation in 28 European countries. Based on Turkey’s strategic geographical location and the energy and environmental degradation challenges faced by Turkey, Saint Akadiri [38] analyzed the links between carbon emissions, electricity consumption, economic growth and globalization in Turkey from 1970 to 2014 by using a variety of econometric techniques.

Differing research purposes and systems of coupling coordination contribute to the diversity of influencing factors. Such factors are helpful for decision-makers to forecast the trend of the relationship between nature-based economy development and environmental protection [32,39]. In terms of these related studies, some discussed the impact of new economic values on tourism transformation [21,40]; some analyzed the factors influencing the causality among tourism, economic growth, carbon dioxide emission, capital formation and energy consumption [5,19,41]; some identified conflicts’ tendency between nature-based tourism development and environment protection [29,30]. Consequently, some scholars carried out studies on factors affecting coupling coordination, such as the ecological footprints of agricultural inputs and agricultural pollutants, terrain conditions, inland waterway mileage, railway passenger volume, inland waterway passenger volume, inland waterway passenger turnover, the proportion of nature reserves in the jurisdiction, per capita water consumption, industrial wastewater discharge and industrial SO_2_ discharge, etc [39,42,43]. According to previous studies, most of these factors were related to human economic activities, and only a few mentioned the limitations of natural conditions. Therefore, this study will consider the natural basis and human activities in the selection of factors.

### 2.3. Analytical Framework of the EETT

An extremely complex interaction exists in the system of EETT. Economic growth promotes environmental protection and traffic and tourism development [44,45]; the ecological environment is the foundation of economic activities and the sustainable development of tourism and traffic infrastructure [5,41]; and tourism promotes economic and traffic development and environmental protection [46]. In addition, the allopatry of tourism activities strengthens the connection between tourism and traffic, and the accessibility of traffic and the spatial movement of tourism are closely related to economic cost and time cost [47,48]; a perfect transport network is conducive to the improvement of unbalanced tourism, ecological environment and economic development [20,26,35]. With the popularity of virtual communities, the ways of tourism information sharing have increased, nowadays both discussion and decision-making process in tourism happens through social networks [48]. However, the developing economy, tourism and traffic may reversely cause environmental hazards [44,45,49]; economic structure, a fragile environment and limited traffic resources will restrict tourism development [12,50,51]; a single tourism industry, transportation industry or environment-dependent industry may negatively affect economic stability [29,52,53,54]; and economic expansion, environmental protection and tourism development will directly affect transportation modernization level [4,20,35]. Obviously, EETT is an organic whole. Thus, the system that is composed of an economic subsystem, an environment subsystem, a tourism subsystem and a traffic subsystem can be defined as a coupling system, in which coordination of the coupling relationship is essential to realize sustainable regional development.

**Figure 1:**
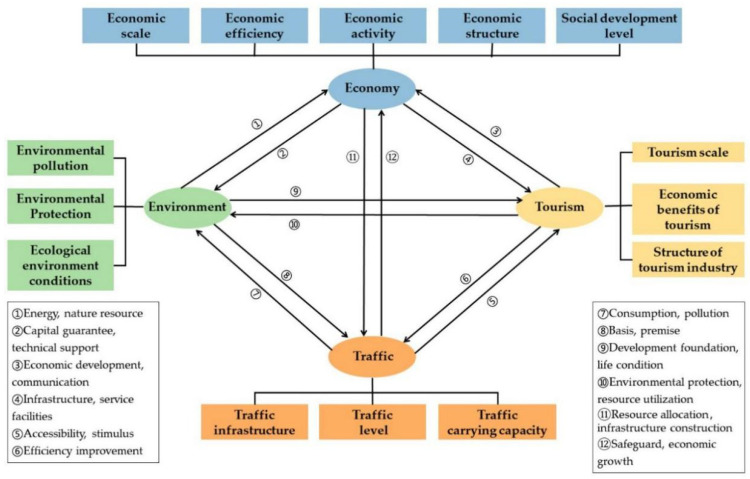
Framework of coupling and coordinated development of EETT. Source: own elaboration.

Based on previous literature, this study attempts to recomb the theoretical framework of coupling and coordinated development of EETT by integrating the four subsystems of economy, environment, tourism and traffic (Figure 1). In the economic subsystem, the economic scale reflects the overall economic level, the economic efficiency reflects the relationship between input and output, the economic activity reflects the level of production and consumption, the economic structure reflects the regional productivity layout and the social development level reflects the degree of economic development. In the environment subsystem, the environmental pollution reflects the environmental damage caused by human production activities, the environmental protection reflects the management of and investment in environmental pollution treatment and the ecological environment conditions reflect the quality and potential of the environment. In the tourism subsystem, the tourism scale reflects the scale of the tourist market, the economic benefits of tourism reflect the economic development level of the tourism industry and the structure of the tourism industry reflects the layout of the tourism industry. In the traffic subsystem, the traffic infrastructure reflects the transportation network construction level, the traffic level reflects the overall traffic economic level and the traffic-carrying capacity reflects the transportation level.

## 3. Materials and Methods

### 3.1. Study Area

**Figure 2:**
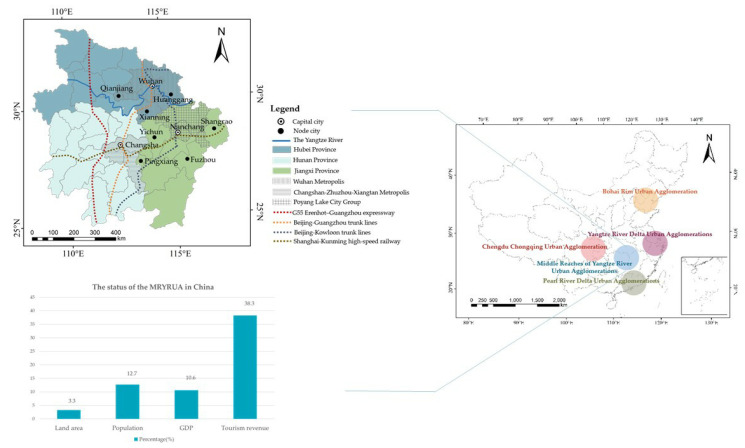
Geographical location of the Middle Reaches of Yangtze River Urban Agglomerations. Data Source: Website of State Bureau of surveying, mapping and geographic information.

The MRYRUA is one of the five national urban agglomerations in China, including Hubei, Hunan and Jiangxi provinces (Figure 2). It is mainly formed by the Wuhan Metropolis, Changsha–Zhuzhou–Xiangtan Metropolis and Poyang Lake City Group. Its area spans over 317,000 km^2^, accounting for 3.3% of the entire land area in China. The MRYRUA is rich in natural resources, such as rivers and lakes, forest resources and cultivated land. The MRYRUA has an important strategic position in the national comprehensive transportation network, which includes railways of the Beijing–Kowloon and Beijing–Guangzhou trunk lines; the Shanghai–Kunming high-speed railway; the G55 Erenhot–Guangzhou expressway; several air routes and some river ports, basically forming a dense three-dimensional transportation network [55,56]. In 2019, the total GDP of the MRYRUA reached CNY 10,476.8 billion, accounting for 10.6% of China’s GDP, and the total population reached 178.2 million, accounting for 12.7% of China’s total population (http://www.stats.gov.cn (accessed on 18 July 2021)). As one of the most developed areas of tourism in China, the MRYRUA is presently undergoing rapid tourism development. In 2019, the MRYRUA received about 2195 million tourists, with tourism revenue of CNY 2537 billion, accounting for 34.8% of all tourists and 38.3% of the tourism revenue of China (https://www.mct.gov.cn/ (accessed on 18 July 2021)). The MRYRUA has become an important part of Chinese tourism development; simultaneously, accommodations, catering, transportation, sightseeing and entertainment have a significant influence on the environment. Correspondingly, a series of impacts of coordinated development imbalances have become important challenges restricting regional development sustainability in the MRYRUA.

### 3.2. Data Sources and Pre-Processing

The required data came mainly from The Yearbook of China Urban Statistics, The Yearbook of China Tourism Statistics, Hubei Statistics Yearbook, Jiangxi Statistics Yearbook, Hunan Statistics Yearbook and Civil Aviation Airport throughput Bulletin. Considering that China’s national economic and social development plan is one cycle every five years, this study chose 1995, 2000, 2005, 2010 and 2017 as the research years. In order to eliminate the influence of dimension and magnitude, the raw data had to be standardized using Formulas (1) and (2) [57,58]. For positive index,

(1) $$X_{ij}^{\prime }=\frac{X_{ij}-\underset{1\leq j\leq n}{\min}X_{ij}}{\underset{1\leq j\leq n}{\max}X_{ij}-\underset{1\leq j\leq n}{\min}X_{ij}}$$

while for negative index,

(2) $$X_{ij}^{\prime }=\frac{\underset{1\leq j\leq n}{\max}X_{ij}-X_{ij}}{\underset{1\leq j\leq n}{\max}X_{ij}-\underset{1\leq j\leq n}{\min}X_{ij}}$$

where $X_{ij}^{\prime }$ and *X_ij_* represent the standardized value and the original value of index *j* in year *i*, respectively; $\underset{1\leq j\leq n}{\max}X_{ij}$ and $\underset{1\leq j\leq n}{\min}X_{ij}$ indicate the maximum and minimum values of index *j* among all years, respectively.

### 3.3. Methods

#### 3.3.1. Information Entropy Weight Method and Evaluation of Subsystems

The information entropy weight method can determine the indexes’ weight by analyzing the correlation degree and information among indexes, and avoids bias caused by subjective influence to a certain extent [59,60]. The steps are as follows:

Calculate the proportion of the index *j* in year _*i*_ (*Y_ij_*):

(3) $$Y_{ij}=X_{ij}^{\prime }/\overset{m}{\underset{i=1}{\sum }}X_{ij}^{\prime }$$

Calculate the information entropy of the index *j* (*e_j_*); if $X_{ij}^{\prime }$ = 0, 0.00001 is substituted for 0 to calculate *e_j_*:

(4) $$e_{j}=-\frac{1}{\ln m}\overset{m}{\underset{i=1}{\sum }}Y_{ij}\times \ln Y_{ij}\;\left(0\leq e_{j}\leq 1\right)$$

Calculate the entropy redundancy (*d_j_*):

*d_j_* = 1 − *e_j_* (5)

Calculate the weight of the index *j* (*w_j_*):

(6) $$w_{j}=d_{j}/\sum_{j=1}^{n}d_{j}$$

where *n* is the number of indexes, and *m* is the number of years.

The subsystem score can be calculated after standardizing the data. The equation for the score of the *t*th subsystem is as follows:

(7) $$S_{t}=\overset{K_{t}}{\underset{j=1}{\sum }}w_{j}X_{ij}^{\prime }\left(i=1,2,3,4\right)$$

where *K_t_*(*t* = 1,2,3,4) is the number of indexes in the *t*th subsystem, and *t* = 1,2,3,4 represent the economic subsystem, environment subsystem, tourism subsystem and traffic subsystem, respectively; *w_j_* is the weight of index *j*, which can be calculated by information entropy weight.

#### 3.3.2. Coupling Coordination Degree Model

The coupling degree model of interaction between two or more systems (or elements) is usually derived from the capacity coupling coefficient model in physics; that is, the coupling degree model of economy, environment, tourism and traffic is deduced from the following [61,62,63]: Cn=[(u1,u2,…um)∏ (ui+uj)]1n (where *u_i_* (*i* = 1, 2, 3, …, *m*) is the comprehensive evaluation function of each subsystem):

(8) $$C={\left[\frac{S_{1}\times S_{2}\times S_{3}\times S_{4}}{{\left(S_{1}+S_{2}+S_{3}+S_{4}\right)}^{4}}\right]}^{\frac{1}{4}}$$

The model is concise and has obvious physical significance. However, when calculating the coupling degree of multiple systems, if the function value of one system is 0, the coupling degree will be 0 regardless of the function value of other systems, which does not conform to the reality of social and economic systems. In addition, the numerical distribution range of the coupling degree calculated by this method is narrow and lacks hierarchy. Therefore, this study attempts to use the coefficient of variation in a statistical sense to deduce a function that is approximately similar to it but can overcome the above problems. Specifically, according to the concept of coupling degree and coupling mechanism, the smaller the coefficient of variation *C*_1_ among *S*_1_, *S*_2_, *S*_3_ and *S*_4_ is, the better:

(9) C1=(S1−A)2+(S2−A)2+(S3−A)2+(S4−A)24A

where $A=\frac{S_{1}+S_{2}+S_{3}+S_{4}}{4}$. By further calculating the formula of *C*_1_, we can obtain the following results:

(10) $$C_{1}=\sqrt{3-\frac{8\times \left(S_{1}\times S_{2}+S_{1}\times S_{3}+S_{1}\times S_{4}+S_{2}\times S_{3}+S_{2}\times S_{4}+S_{3}\times S_{4}\right)}{{\left(S_{1}+S_{2}+S_{3}+S_{4}\right)}^{2}}}$$

The necessary and sufficient condition for *C*_1_ to be smaller is that the larger the value of *C*_2_ is, the better.

(11) $$C_{2}=\frac{8\times \left(S_{1}\times S_{2}+S_{1}\times S_{3}+S_{1}\times S_{4}+S_{2}\times S_{3}+S_{2}\times S_{4}+S_{3}\times S_{4}\right)}{{\left(S_{1}+S_{2}+S_{3}+S_{4}\right)}^{2}}$$

In general, 0 ≤ C_1_ ≤ 1 and 2 ≤ C_2_ ≤ 3, so the following functions are constructed:

(12) $$C_{3}=\frac{8\times \left(S_{1}\times S_{2}+S_{1}\times S_{3}+S_{1}\times S_{4}+S_{2}\times S_{3}+S_{2}\times S_{4}+S_{3}\times S_{4}\right)}{{\left(S_{1}+S_{2}+S_{3}+S_{4}\right)}^{2}}-2$$

The value of the function is between [0, 1], and the larger the value is, the smaller the dispersion degree is and the higher the coupling degree is. Furthermore, the function can be simplified as follows:

(13) $$C_{4}=2-\frac{4\times \left(S_{1}{}^{2}+S_{2}{}^{2}+S_{3}{}^{2}+S_{4}{}^{2}\right)}{{\left(S_{1}+S_{2}+S_{3}+S_{4}\right)}^{2}}$$

Furthermore, in order to find the function value—that is, make the coupling degree more hierarchical—the development coupling degree calculation model is given:

(14) $$C=\sqrt{2-\frac{4\times \left(S_{1}{}^{2}+S_{2}{}^{2}+S_{3}{}^{2}+S_{4}{}^{2}\right)}{{\left(S_{1}+S_{2}+S_{3}+S_{4}\right)}^{2}}}$$

The numerical value of the function is also between [0, 1], and the larger the value is, the smaller the dispersion degree is and the higher the coupling degree is. It can better reflect the coupling degree of the economic, environment, tourism and traffic development levels, and can effectively avoid the measurement problem when some index values are 0. When *S*_1_, *S*_2_, *S*_3_ and *S*_4_ are equal values other than 0, the coupling degree between the four subsystems is the highest when *C* = 1; when all four are 1, the system reaches benign resonance coupling; and when *C* = 0, the four subsystems are completely independent.

In order to evaluate the coordination degree of EETT interaction better, the coordination degree model was introduced to consider the interaction strength of the subsystems and the development level of subsystems. The calculation formula is as follows:

(15) $$D=\sqrt{C\times T}$$

(16) $$T=\frac{\left[S_{1}+S_{2}+S_{3}+S_{4}\right]}{4}$$

where *D* represents the degree of coupling coordination, and *D*∈[0, 1]; *C* represents the degree of coupling among the four subsystems; *T* reflects the overall level of four subsystems.

The degree of coupling coordination (*D*) can be categorized into 10 subtypes [53,54,63]: extremely uncoordinated, seriously uncoordinated, moderately uncoordinated, mildly uncoordinated, on the verge of uncoordinated, barely coordinated, basically coordinated, fairly coordinated, favorably coordinated and highly coordinated (Table 1).

#### 3.3.3. Exploratory Spatial Data Analysis Model

In this study, exploratory spatial data analysis is used to explain the spatial dependence, spatial association and autocorrelation phenomena related to the spatial location of 42 cities. According to the literature review, in addition to the information entropy weight of exploratory spatial data analysis, the commonly used quantitative indexes include the Global Moran index and the Local Moran index, which are used for global–spatial autocorrelation analysis and local–spatial autocorrelation analysis, respectively [64,65,66]. The exponential model is as follows:

(17) $$I=\frac{n}{V}\frac{\overset{n}{\underset{i=1}{\sum }}\overset{n}{\underset{j=1}{\sum }}v_{ij}\left(x_{i}-\bar{x}\right)\left(x_{j}-\bar{x}\right)}{\overset{n}{\underset{i=1}{\sum }}{\left(x_{i}-\bar{x}\right)}^{2}}$$

where *n* is the number of spatial units indexed by *i* and *j*; *x* refers to the variable; $\bar{x}$ denotes the mean of *x*; *v_ij_* is a matrix of spatial weights of units *i* and *j* with zeroes on the diagonal; and *V* is the sum of all *v_ij_*. The value of *I* ranges from −1 to 1. When the value of *I* is lower than zero, the spatial autocorrelation is negative. The larger the absolute value of *I* is, the stronger the spatial autocorrelation is. When the value is equal to zero, it indicates spatial randomness.

The local–spatial autocorrelation index can measure the local–spatial association between each region and its surrounding areas. Combined with a Moran scatter map or scatter map, the spatial structure of local differences can be visualized and the spatial distribution law can be studied. The Local Moran’s I (LISA) statistic is generally used to measure the autocorrelation of local space. The model is specified as follows:

(18) $$I_{i}=\frac{n\left(x_{i}-\bar{x}\right)}{\overset{n}{\underset{i=1}{\sum }}{\left(x_{i}-\bar{x}\right)}^{2}}\overset{n}{\underset{j=1}{\sum }}v_{ij}\left(x_{j}-\bar{x}\right)$$

The Global Moran’s I index measures the similarity between high and low coupling coordination levels, but only reflects the aggregation or dispersion degree of the spatial distribution of the coupling coordination degree among cities. In local autocorrelation analysis, there are four types of spatial association: High–High type, High–Low type, Low–High type and Low–Low type, which reflect the local–spatial aggregation of high or low values of observations.

#### 3.3.4. Grey Correlation Degree Analysis

This study intended to explore the factors influencing the coupling coordination degree of cities in the MRYRUA from the perspective of natural factors and human activities [32,39,42]. In terms of natural factors, vegetation index (f_1_), average elevation (f_2_), average temperature (f_3_) and annual average precipitation (f_4_) are used to reflect urban precipitation conditions, urban temperature, geographical conditions and natural environment, which are the basic elements of urban sustainable development. In terms of human activities, population density (f_5_), natural population growth rate (f_6_), local general public budget expenditure (f_7_), land used for urban construction as a percentage of the urban area (f_8_), number of operating buses per square meter of road (f_9_) and number of operating taxis per square meter of road (f_10_) are used to reflect the level of resource allocation and infrastructure construction, which are the basic elements of urban coordinated development.

In order to avoid the error of data on a certain time section, the dependent variables and explanatory variables were calculated by taking the average value of five years. The method can be divided by 4 steps [33]: Firstly, the data column of the degree of coupling coordination is specified as noted *D*_0_(*k*).

Secondly, the data initial processing and dimensionless treatment are calculated:

(19) $$f_{a}=\frac{l\left(k\right)}{l_{1}\left(1\right)}\;a=0,1,2,3,4\ldots \ldots$$

Thirdly, the absolute difference value of *D*_0_ with *f_a_* is calculated; then, the correlation coefficient $\varepsilon_{a}$(*k*) is computed by the following equation:

(20) $$\varepsilon_{a}\left(k\right)=\frac{\underset{a}{\min}\underset{k}{\min}\left[D_{0}\left(k\right)-f_{a}\left(k\right)\right]+\rho \;\underset{a}{\max}\underset{k}{\max}\left[D_{0}\left(k\right)-f_{a}\left(k\right)\right]}{\left[D_{0}\left(k\right)-f_{a}\left(k\right)\right]+\rho \;\underset{a}{\max}\underset{k}{\max}\left[D_{0}\left(k\right)-f_{a}\left(k\right)\right]}$$

According to Formula (20), the value of ρ determines the contribution of $\underset{a}{\max}\underset{k}{\max}\left[D_{0}\left(k\right)-f_{a}\left(k\right)\right]\;\mathrm{to}\;\varepsilon_{a}$(*k*)—that is, the influence of other sequences on the reference sequence and comparison sequence participating in the comparison—which is the embodiment of the systematic integrity of the correlation degree. This effect of ρ will further affect the size of the distribution interval of the correlation degree and will thus affect the results of the correlation analysis. In practice, not only the ranking of the correlation degree but also the distribution range of the correlation degree should be considered. If the distribution interval of the correlation degree is very small, even if the ranking of the correlation degree can be obtained, the similarity between sequences cannot be clearly distinguished due to the small difference between correlation degrees. Therefore, in order to achieve a good discrimination effect, the distribution range of the correlation degree must be made to be the largest while obtaining the order of the correlation degree. The following shows the influence of the value of ρ on the distribution range of the correlation degree from the perspective of whether the sequence is stable.

Through the initialization of the data, make $\underset{a}{\min}\underset{k}{\min}\left[D_{0}\left(k\right)-f_{a}\left(k\right)\right]=0$; then, Formula (20) can be changed into the following:

(21) $$\varepsilon_{a}\left(k\right)=\frac{\rho \;\underset{a}{\max}\underset{k}{\max}\left[D_{0}\left(k\right)-f_{a}\left(k\right)\right]}{\left[D_{0}\left(k\right)-f_{a}\left(k\right)\right]+\rho \;\underset{a}{\max}\underset{k}{\max}\left[D_{0}\left(k\right)-f_{a}\left(k\right)\right]}$$

(22) $$\theta_{a}\left(k\right)=\frac{D_{0}\left(k\right)-f_{a}\left(k\right)}{\underset{a}{\max}\underset{k}{\max}\left(k\right)}$$

Then, Formula (21) becomes

(23) $$\varepsilon_{a}\left(k\right)=\frac{\rho }{\theta_{a}\left(k\right)+\rho }$$

When the sequence is not stable, it often appears that $\underset{a}{\max}\underset{k}{\max}\left[D_{0}\left(k\right)-f_{a}\left(k\right)\right]>D_{0}\left(k\right)-f_{a}\left(k\right)\left(\underset{a}{\max}\underset{k}{\max}\left[D_{0}\left(k\right)-f_{a}\left(k\right)\right]\neq D_{0}\left(k\right)-f_{a}\left(k\right)\right)$; then, $\theta_{a}\left(k\right)$ is very small. If ρ is larger—for example, ρ = 1—then $\varepsilon_{a}\left(k\right)$ is completely dominated, and the value of $\varepsilon_{a}\left(k\right)$ is close to 1. Therefore, the correlation degree obtained is very close, and the distribution range of the correlation degree is very small. It is difficult to distinguish the similarity between the reference sequence and the comparison sequence. Therefore, in practical applications, the value of ρ should be reduced as much as possible so as to weaken the dominance of $\underset{a}{\max}\underset{k}{\max}\left[D_{0}\left(k\right)-f_{a}\left(k\right)\right]$ over $\varepsilon_{a}\left(k\right)$. On the other hand, when the sequence is very stable, the difference between $\underset{a}{\max}\underset{k}{\max}\left[D_{0}\left(k\right)-f_{a}\left(k\right)\right]$ and $D_{0}\left(k\right)-f_{a}\left(k\right)$ is not very big. At this time,$\;\theta_{a}\left(k\right)$ is large. If ρ is a small value, then $\theta_{a}\left(k\right)$ is small and is also very close. Therefore, the value of the correlation degree will be very close and the distribution interval will be very small, making it difficult to distinguish the similarity between the reference sequence and the comparison sequence. Therefore, in this case, ρ should be larger, and the effect of $\underset{a}{\max}\underset{k}{\max}\left[D_{0}\left(k\right)-f_{a}\left(k\right)\right]$ on $\varepsilon_{a}\left(k\right)$ should be increased to fully reflect the integrity of the correlation degree.

As ρ∈ (0, 1], the boundary is defined by ρ = 0.5. In this study, we take ρ = 0.5; thus, the calculation formula is as follows:

(24) $$\varepsilon_{a}\left(k\right)=\frac{\underset{a}{\min}\underset{k}{\min}\left[D_{0}\left(k\right)-f_{a}\left(k\right)\right]+0.5\underset{a}{\max}\underset{k}{\max}\left[D_{0}\left(k\right)-f_{a}\left(k\right)\right]}{\left[D_{0}\left(k\right)-f_{a}\left(k\right)\right]+0.5\underset{a}{\max}\underset{k}{\max}\left[D_{0}\left(k\right)-f_{a}\left(k\right)\right]}$$

Finally, calculate the average correlation coefficient and the grey correlation degree:

(25) $$r_{a}=\frac{1}{N}\sum_{k=1}^{N}\varepsilon_{a}\left(k\right)$$

#### 3.3.5. Index System

Academics have established considerable index systems to evaluate the level of coordination development [29,30]. Most previous studies concentrate more on the dynamic changes of the coordination development of any two or three subsystems, yet there is still a lack of analysis of the whole EETT system and the influencing factors of coupling coordination development. In order to introduce an aggregated index system to evaluate the EETT system, a preliminary determination of the indexes of the EETT system was conducted on the basis of the framework of coupling and coordinated development of EETT (Figure 1) and related studies. Then, focusing on the four cores of economy, environment, tourism and traffic, starting from 14 aspects such as economic scale, economic efficiency, economic activity, economic structure and social development level, etc., the critical indicators were further selected in light of data availability, indicator representativeness and system relevance. Finally, an aggregated index system consisting of four subsystems, 14 aspects and 37 indicators was formulated (shown in Table 2). The index system, direction of indicators, calculation results of index weight and index references are shown in Table 2.

## 4. Results

### 4.1. Coupling Coordination Degree

**Figure 3:**
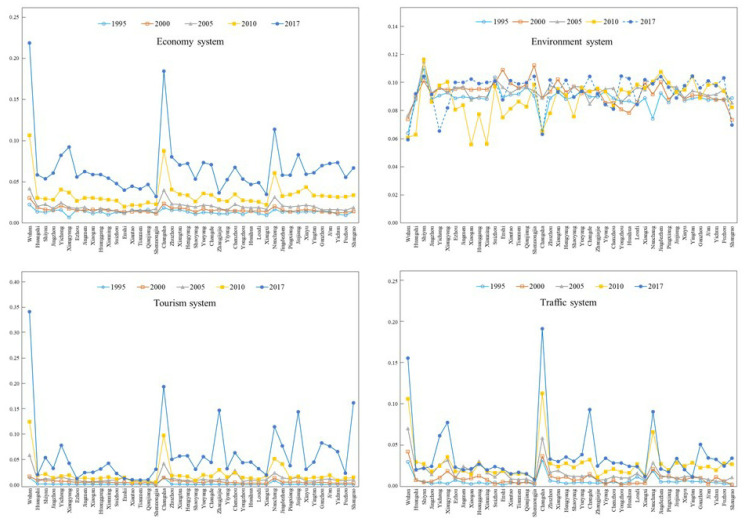
Subsystem coordination degree in MRYRUA from 1995 to 2017. Source: own elaboration.

It can be observed from Figure 3 that the economy subsystem and the tourism subsystem showed a steady growth trend from 1995 to 2017. In 1995, the coordination degree of the tourism subsystem in Wuhan was the highest, and that of Nanchang and Changsha was the lowest. The difference in the environment subsystem between 2010 and 2017 was obvious. While the traffic subsystem in each city was growing steadily, the annual differences were significant.

**Figure 4:**
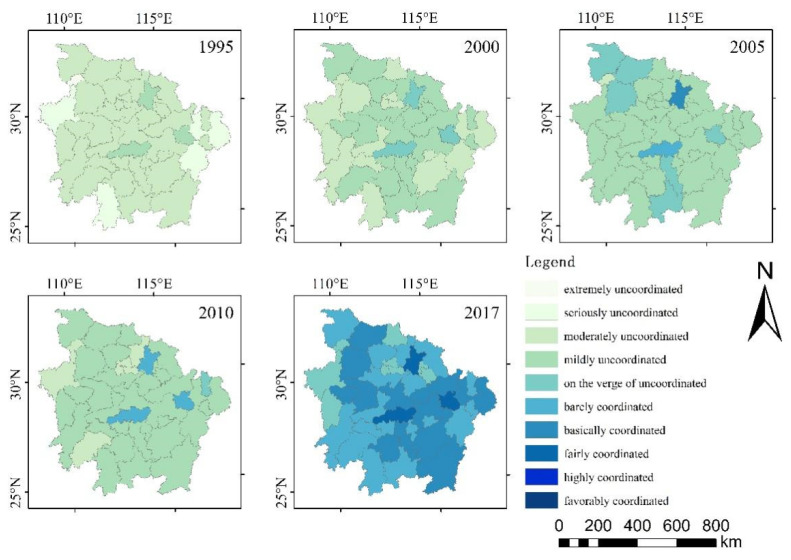
Coupling coordination degree of EETT in MRYRUA from 1995 to 2017. Source: own elaboration.

As illustrated in Figure 4, the degree of coupling coordination of EETT gradually increased from 1995 to 2017, especially in three provincial capitals—Wuhan, Changsha and Nanchang. Overall, cities with a relatively high degree of coupling coordination gradually spread from the center to the south. Based on the degree of coordinated coupling, this study analyzed the development of coupling coordination in three periods. In the uncoordinated period (1995–2000), the degree of coupling coordination of EETT centered at moderately uncoordinated in 1995, and then centered at mildly uncoordinated in 2000. Meanwhile, the coordinated development level increased from extremely and seriously uncoordinated to moderately and mildly uncoordinated, and then on the verge of uncoordinated. During this period, the four subsystems were in a state of uncoordinated development. In the transitional period (2005–2010), the degree of coupling coordination of EETT continued to grow, with the level centered at mildly uncoordinated. While the moderately uncoordinated status was reduced, it moved to barely and basically coordinated. The whole system then reached a status of coordinated development from uncoordinated development. In the coordinated period (2017), significant growth occurred, resulting in the degree of coupling coordination of EETT being mostly centered at barely and basically coordinated. Additionally, in 2017, a state of being on the verge of uncoordinated still existed with the growing status of fairly coordinated.

### 4.2. Spatial Disparity of Coupling Coordination Degree

**Figure 5:**
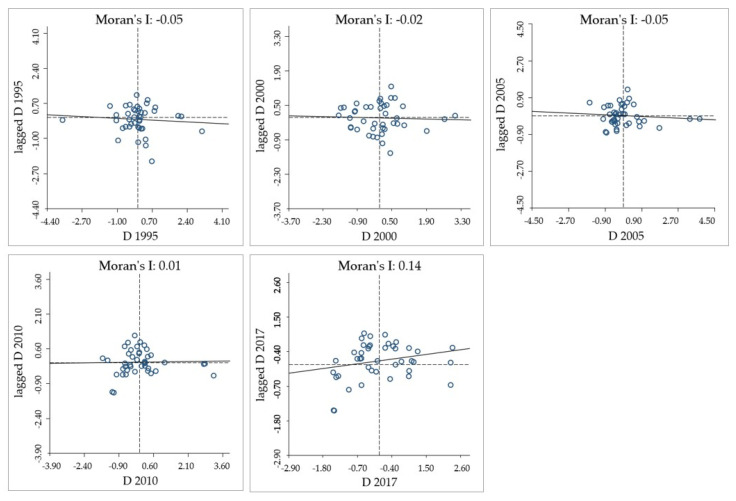
Moran scatter plot of coupling coordination degree from 1995 to 2017. Source: own elaboration. Note: “lagged D” means spatial lagging factors.

It can be seen from Figure 5 that since 2010, the coupling coordination degree of EETT in the MRYRUA presents a positive correlation; that is, it has obvious spatial agglomeration, and the degree of agglomeration has an upward trend year by year.

**Figure 6:**
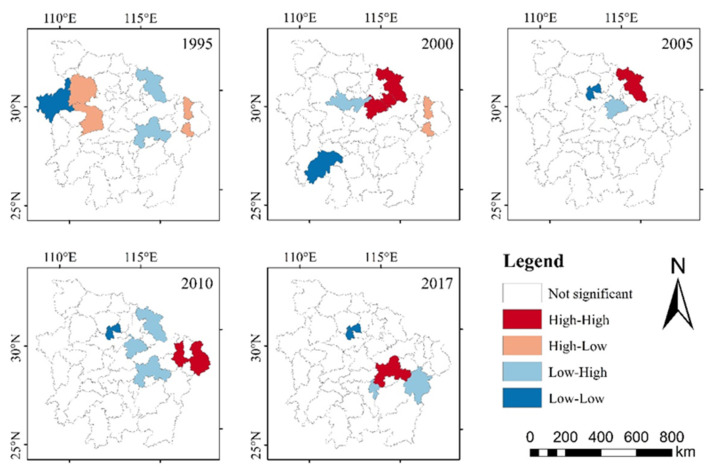
Local indicators of spatial association (LISA) cluster maps for coupling coordination degree. Source: own elaboration.

The dynamic spatial agglomeration of the coupling coordination degree in the MRYRUA during 1995–2017 is illustrated in Figure 6. Spatially, the coupling coordination degree of EETT in the MRYRUA was discrete in 1995, 2000 and 2005. In 2010, most cities were not significant; Shangrao was the “High–High” type, Qianjiang was the “Low–Low” type and the “Low–High” types were Huanggang, Xianning and Yichun. In 2017, Yichun was the “High–High” type, Qianjiang was still the “Low–Low” type and the “Low–High” types were Pingxiang and Fuzhou.

From 2010 to 2017, Qianjiang, as the pole of “Low–Low” agglomeration, did not change. The scope of the surrounding areas was gradually expanded, and the coupling coordination degree of Qianjiang improved slowly, which indicated that there was no city with a positive radiation effect around Qianjiang; the “High–High” concentration area was transferred from the east to the south, and Yichun changed from “Low–High” to “High–High”, having been positively affected by the surrounding “High–High” concentration area; the “Low–High” concentration area also transferred from the east to the south, indicating that Yichun has also driven the coupling coordination development of adjacent regions.

### 4.3. Influencing Factors

As demonstrated in Table 3, the correlation degree between the degree of coupling coordination and the influencing factors is above 0.5, except for the number of operating taxis per square meter of road (f_10_). It can be asserted that there is a close relationship between the degree of coupling coordination and the influencing factors. Furthermore, the average degree shows that the grey correlation degree between the coupling coordination degree and natural factors is higher than that with human factors.

In terms of the ten influencing factors, the correlation degree of land used for urban construction as a percentage of the urban area (f_8_) has the highest value in Table 3, and the degree of the vegetation index (f_1_) is close to the degree of f_8_. It means that “land used for urban construction as a percentage of urban area” and “vegetation index” affect the degree of coupling coordination most strongly in contrast to the rest of the natural and human factors. The correlation degree of average temperature (f_3_) is the third-largest, which shows that the average temperature has a significant effect on the degree of coupling coordination. Among the remaining seven indexes, the most important ones are annual average precipitation (f_4_), natural population growth rate (f_6_), number of operating buses per square meter of road (f_9_), local general public budget expenditure (f_7_), population density (f_5_) and average elevation (f_2_). However, as the only factor with a correlation degree of below 0.5, the number of operating taxis per square meter of road (f_10_) has less influence on the coupling coordination degree compared with other factors.

## 5. Discussion

### 5.1. Dynamic Change of Coupling Coordination Degree in MRYRUA

During the study period, there was an increasing overall trend of the coupling coordination degree of the MRYRUA. By comparison with the findings of Tang [29] and Cheng et al. [27], due to the limitation of geographical space, the cities with a relatively low coupling coordination degree were concentrated in the mountainous and hilly areas in the west and north, which is consistent with the findings of this study. Chen et al. [18] found that with the implementation of the central traffic integration strategy and the ecological environment protection plan of the Yangtze River Economic Belt, the traffic carrying capacity and accessibility of each city have improved, and the ecological environment has also improved. Consequently, although the coupling coordination degree of MRYRUA improved during the study period, the traffic in hilly areas was relatively backward, the development was relatively backward, and the coupling coordination degree grew slowly. In the uncoordinated period (1995–2000), the degree of coupling coordination of EETT turned from moderately uncoordinated to mildly uncoordinated, and the whole was still uncoordinated. In the transitional period (2005–2010), the degree of coupling coordination of EETT continued to grow, the whole system reached a status of coordinated development from uncoordinated development. In the coordinated period (2017), significant growth occurred, resulting in the degree of coupling coordination of EETT being mostly centered at barely and basically coordinated. Additionally, in 2017, a state of being on the verge of uncoordinated still existed with the growing status of fairly coordinated.

Compared with previous research [67,68,69,70], this study focuses on the observation of the three provincial capital cities, and finds that during the research period, the growth rate of coupling coordination degree of the three capital cities was significantly higher than that of other cities, while the growth rate of coupling coordination degree of Nanchang was significantly higher than that of Wuhan and Changsha. With the promotion of “ecological civilization” and the advent of the “high-speed rail era” [6,71], the contradictions among environmental protection, economic development, traffic system improvement and tourism development are gradually weakening. Especially in provincial capital cities, resources and industries are more concentrated, and policies are first implemented there, so the degree of coordinated development is more obvious. Since 2000, Nanchang has become a transit hub for commerce and trade in coastal areas to radiate to the central and western regions, thus driving the economic and traffic growth of the surrounding areas. Therefore, Nanchang has steadily increased from moderately uncoordinated in 1995 to fairly coordinated in 2017.

### 5.2. Spatial Agglomeration of Coupling Coordination Degree

The spatial pattern is explored by global–spatial autocorrelation analysis and local–spatial autocorrelation analysis, which play an important role in analyzing the correlation between coupling coordination degree and spatial position [72]. From 1995 to 2010, the coupling coordination development of EETT in the MRYRUA was in a discrete state. With the integrated development of the MRYRUA, the implementation of the strategy of “rise of Central China” [13] and the construction of intercity rail transit [14], the relationship between cities was gradually strengthened, and the agglomeration phenomenon began to appear in 2010. From 2010 to 2017, most cities were not significant because they were basically in the transition zone between a high and low degree of coupling coordination development. They were neither affected by the spillover nor had a driving effect on other regions, so they were in the dilemma of advance and retreat. With time, the degree of coupling coordination improved. The “Low–Low” agglomeration areas remained unchanged, while the “High–High” and “Low–High” agglomeration regions spread from the east to the south, which was the result of the joint action of macro-policies and natural resources’ conditions.

From 2010 to 2017, Qianjiang, as the pole of “Low–Low” agglomeration, did not change. On the contrary, Yichun changed from “Low–High” to “High–High”, having been positively affected by the surrounding “High–High” concentration area. Qianjiang is an important node city of Hubei Province’s “Two Circles and Two Belts” strategy [58,72]. Due to the lack of outstanding advantages in natural conditions and the influence of Wuhan’s siphon effect, the coupling coordination degree of EETT was not significantly increased, so Qianjiang was not affected by the highly coordinated area. Yichun is a new industrial city in Jiangxi Province, with rich natural and cultural resources and a developed transportation network. Yichun has been awarded many honorary titles, such as “Livable city in China”, “World famous cultural tourism city”, “National garden city”, etc. Therefore, Yichun itself has positively affected and promoted the coupling coordination development of adjacent regions.

### 5.3. Identification of Influencing Indicators

The identification and ranking of the influencing factors of coupling coordination degree by grey correlation degree analysis are not only conducive to the in-depth understanding of the limiting factors and driving mechanism of regional development, but also provide a decision-making basis for the future economic and transportation network structure adjustment, ecological environment protection and tourism industry development of urban agglomeration [39,55]. During the study period, compared with human factors, natural factors had more influence on the coupling coordination degree. In particular, the influence of vegetation index, average temperature and annual average precipitation as natural factors on the coupling coordination degree was relatively strong. Previous studies have illustrated that agricultural pollutants, terrain conditions, inland waterway mileage and railway passenger volume have an impact on coupling coordinated development [32,33,39,42,43], which are analyzed from the perspective of a single factor. In this study, many human factors are concerned, among which land used for urban construction as a percentage of urban area, natural population growth rate and number of operating buses per square meter of road are most important human factors.

This suggests that the natural factors, as well as the combined effects of natural factors and human factors, should not be ignored. During the study period, cities with superior geographical and climatic conditions and rich resources have great development potential and rapid growth of coupling coordination degree, for example, Wuhan, Nanchang and Yichun. Land used for urban construction as a percentage of urban area is the basis of social benefits. It is clear that the human–land relationship was one key aspect for achieving regional coordinated development [73]. Results of this study confirm that vegetation quantity, geographical location, sunshine duration and air humidity may be important reasons for influencing factors of regional development. City planners and government organizations should pay more attention to the influence of natural factors while they design regional development policy. Additionally, the natural population growth rate and number of operating buses per square meter of road had noticeable effects on the coupling coordination degree of EETT. Traffic and economic problems caused by the aging of the population and the shortage of public transportation may be significant reasons for restricted economic development.

### 5.4. Policy Implications

Understanding the spatial relationship and influencing factors of the degree of coupling coordination between cities can provide a scientific reference for the coordinated development of urban agglomerations. The findings of this study demonstrate that the relationship between cities is extremely complex, with repression and synergies. On the other hand, coupling and coordinated development is influenced by both natural and human factors. For example, the complexity of terrain and low natural population growth rate will lead to the difficulty of traffic access and slow economic growth [51,74]. Rich natural resources, pleasant climate conditions and reasonable land-use layout can promote the development of tourism and economy, thus promoting environmental protection and traffic development [20,26,75]. Therefore, the development of MRYRUA should focus on the balance between core and edge. The coordinated development of Wuhan, Changsha, Nanchang, and Yichun is better, they need to continue to enhance their strength and radiate the surrounding areas, but the “edge area” needs more attention and support. In addition, urban agglomerations are usually traffic centers, so it is necessary to plan routes reasonably to make high-speed rail lines cover as many areas as possible. At the same time, tourism routes should be added to cover areas with rich tourism resources but less developed transportation, and cooperation with other airlines should be strengthened to reduce costs and pollution.

### 5.5. Limitations

The results are objective and credible, and they break through the limitations of having only a single province or city as the analysis unit and assessing the coupling relationship between two or three subsystems as the research content. Furthermore, the results could help identify contributors in the complicated coupling relationship of EETT and understand the spatial variation characteristics, thus allowing the implementation of regional development strategies to better balance economic growth, environmental conservation, tourism growth and traffic development. However, the assessment results are constrained by the data acquisition technique, so the established coupling coordination evaluation system fails to fully reflect the development of the four subsystems in each city. In addition, more influencing indicators should be considered when performing a grey correlation degree analysis. Consequently, further studies are essential to consider more influencing factors and develop the spatial analysis of the EETT system in the future.

## 6. Conclusions

Taking MRYRUA as an example, this study discussed the coupling and coordination relationship among the four subsystems of economy, environment, tourism and traffic, explored the spatial pattern of coupling coordination degree of EETT, analyzed influencing factors of coupling coordination degree, and discussed the internal logic of coupling coordination degree of EETT to promote regional sustainable development, which laid a foundation for understanding EETT system. It also provided inspiration for sustainable regional development and coordinated development of urban agglomerations. The conclusions are as follows:

(1) The coupling coordination degree of EETT transitioned from the uncoordinated period to the coordinated period; the coupling coordination degree of each city kept increasing, accompanied by a phenomenon where some cities’ degree decreased first and then increased, which was basically consistent with the research results in the previous literature [27,29]. Particularly during the study period, the growth rate of provincial capital cities is greater, which indicates that the concentration of resources, the scale of industry and the radiation of transportation provide an important guarantee for the coordinated development of EETT.
(2) The results of the global–spatial autocorrelation analysis and local–spatial autocorrelation analysis documented that the spatial agglomeration degree in the MRYRUA has shown a positive trend since 2010. “High–High” and “Low–High” agglomeration regions spread from the east to the south; in particular, Qianjiang has consistently been a “Low–Low” agglomeration area, while Yichun changed from “Low–High” to “High–High”. The adjustment of national and regional development strategies plays an important role in the coordinated development of the EETT system, especially the policy adjustment of industrial transformation and upgrading.
(3) Of the natural factors, “vegetation index”, “average temperature” and “annual average precipitation” are the most significant influencing factors. Regarding the human factors, “land used for urban construction as a percentage of urban area”, “natural population growth rate” and “number of operating buses per square meter of road” have a great impact on the coupling coordination degree. The identification results of human factors are consistent with relevant literature [30,39,43], and moreover, this study comprehensively considers the combined effects of natural and human factors, where the influence of natural factors on the coupling coordination degree is higher than that of human factors.

## Figures and Tables

**Table 1:** Metrics for the coordinated development level.

Range	(0, 0.1)	(0.1, 0.2)	(0.2, 0.3)	(0.3, 0.4)	(0.4, 0.5)
**category**	extremely uncoordinated	seriously uncoordinated	moderately uncoordinated	mildly uncoordinated	on the verge of uncoordinated
**Range**	**(0.5, 0.6)**	**(0.6, 0.7)**	**(0.7, 0.8)**	**(0.8, 0.9)**	**(0.9, 1)**
**category**	barely coordinated	basically coordinated	fairly coordinated	favorably coordinated	highly coordinated

**Table 2:** Evaluation indicator system for EETT. Data source: The Yearbook of China Urban Statistics, The Yearbook of China Tourism Statistics, Hubei Statistics Yearbook, Jiangxi Statistics Yearbook, Hunan Statistics Yearbook and Civil Aviation Airport throughput Bulletin.

Subsystem	First-Class Index	Second-Class Index	Direction	Weights	References
Economy	Economic scale	GDP	+	0.034	[45,46,47]
		GDP per capita	+	0.021	[45,46,47]
		Regional fiscal revenue	+	0.048	[47,48,49]
		Fixed-asset investment	+	0.044	[47,48,50]
	Economic efficiency	Output-to-input ratio	+	0.021	[46,47,48,51]
	Economic activity	GDP growth rate	+	0.001	[45,46,47]
		Total retail sales of consumer goods	+	0.037	[47,48,51]
	Economic structure	The ratio of the added value of the primary industry to GDP	−	0.007	[45,46,47]
		The ratio of the added value of the secondary industry to GDP	−	0.001	[45,46,47]
		The ratio of the added value of the tertiary industry to GDP	+	0.001	[45,46,47]
	Social development level	Per capita disposable income of urban residents	+	0.010	[46,47,51]
		Per capita disposable income of rural residents	+	0.015	[47,48]
		Urban population unemployment rate	−	0.008	[48,52]
Environment	Environmental pollution	Total discharge of industrial wastewater	−	0.020	[48,50,53]
		Total emission by industries	−	0.038	[48,50,54]
		Total amount of industrial solid waste	−	0.028	[47,49]
	Environmental protection	Urban sewage treatment rate	+	0.013	[46,48,50]
		Life garbage treatment rate	+	0.004	[48,50,51]
		Comprehensive utilization rate of industrial solid waste	+	0.003	[46,50]
	Ecological environment conditions	Per capita green area	+	0.023	[47,48]
		Built-up area green coverage rate	+	0.002	[47,49,54]
Tourism	Tourism scale	Number of total tourists	+	0.046	[31,45,52]
		Number of domestic tourists	+	0.046	[45,55]
		Number of international tourists	+	0.061	[45,52,54]
	Economic benefits of tourism	Total tourism revenue	+	0.058	[31,55,56]
		Domestic tourism revenue	+	0.059	[31,56]
		International tourism revenue	+	0.071	[52,54,56]
	Structure of tourism industry	Number of travel agencies	+	0.016	[45,55]
		Number of star-rated hotels	+	0.013	[45,52,56]
		Number of tourist enterprises	+	0.011	[52,54]
Traffic	Traffic infrastructure	Urban per capita road area	+	0.017	[18,45]
	Traffic level	Per capita freight volume	+	0.010	[17,18,19]
		Per capita passenger volume	+	0.015	[17,19,45]
	Traffic carrying capacity	Number of civilian cars	+	0.034	[18,19,45]
		Number of passenger cars	+	0.042	[17,18,45]
		Number of other cars	+	0.022	[18,45]
		Take-offs and landings of aircrafts	+	0.100	[19,45]

**Table 3:** Results of grey correlation degree analysis.

Factors	f_1_	f_2_	f_3_	f_4_	f_5_	f_6_	f_7_	f_8_	f_9_	f_10_
Grey correlation degree	0.82	0.53	0.75	0.67	0.56	0.67	0.59	0.84	0.61	0.48
Average degree	0.70				0.62					
Sorting	2	8	3	4	7	4	6	1	5	9

## Data Availability

Not applicable.
